# Murine Typhus in Children, South Texas^1^

**DOI:** 10.3201/eid1306.061566

**Published:** 2007-06

**Authors:** Kevin Purcell, Jaime Fergie, Kevin Richman, Lisa Rocha

**Affiliations:** * Healthcare Leaders 2B/Pediatric Research 4U, Corpus Christi, Texas, USA; † Texas A&M University College of Medicine, College Station, Texas, USA; ‡ Driscoll Children’s Hospital, Corpus Christi, Texas, USA

**Keywords:** Seroprevalence, Rickettsia typhi, murine typhus, children, dispatch

## Abstract

Children from South Texas were evaluated for immunoglobulin G to *Rickettsia typhi,* the causative agent of murine typhus. Of 513 children, 8.6% of those 1–5 years of age, 13.3% of those 6–11 years of age, and 13.8% of those 12–17 years of age had positive results.

*Rickettsia typhi* causes murine typhus in humans, a febrile illness with headache and rash. Despite the disappearance of *R. typhi* from most of the United States, 9–72 cases per year of murine typhus were reported to the Texas Department of State Health Services from 1994 to 2003 (*1*). An earlier study of 200 cases reported in Texas from 1980 through 1984 found that 29% of the patients resided in Nueces County (*2*). Children with murine typhus often have nonspecific signs and symptoms that mimic those of common viral infections, and the illness usually resolves without antimicrobial drug therapy (*3*,*4*). We believe that many cases go unrecognized and unreported. The objective of this study was to determine the seroprevalence of *R. typhi* in children residing in Nueces County and to assess whether the seroprevalence increases with age due to a greater chance of exposure over time.

## The Study

Driscoll Children’s Hospital is a tertiary care pediatric teaching hospital located in Corpus Christi, Texas, the seat of Nueces County. A convenience sample of serum residuals was obtained from blood samples of children seen in the hospital, its clinic, and its emergency department. Serum was evaluated by use of an indirect immunofluorescence antibody (IFA) test kit for immunoglobulin G (IgG) to *R. typhi* and *R. rickettsii* (Focus Diagnostics, Cypress, CA, USA). Specimen preparation, testing, quality control, and interpretation were done as described in the package insert (www.focusdx.com). Reciprocal titers >64 were considered positive. Endpoint titers were not determined. Testing was performed by the laboratory technician who does all rickettsia assays for the hospital.

No patient information was collected other than age, sex, and county of residence. Only serum residuals from children residing in Nueces County who were 1 to 17 years of age were included. A minimum of 150 serum residuals were obtained from children in each of 3 age groups (1–5, 6–11, and 12–17 years), with ≈50% from each age group being boys. χ^2^ analysis and the Fisher exact test were used to compare frequencies between groups. The Institutional Review Board at Driscoll Children’s Hospital approved this research project; informed consent was not required.

Samples (n = 513) were obtained between May 1, 2005, and August 31, 2006; 47.2% were from boys. There were 152 samples from children 1–5 years of age (mean age 2.7 years; 75 boys), 180 from children 6–11 years of age (mean age 8.2 years; 91 boys), and 181 from children 12–17 years of age (mean age 14.4 years; 76 boys).

Of the 152 samples from children 1–5 years of age, 13 (8.6%) were positive for *R. typhi* IgG and 6 (3.9%) were positive for *R. rickettsii* IgG. Four (67%) of the 6 patient samples positive for *R. rickettsii* IgG were also positive for *R. typhi* IgG. Of the 180 samples from children 6–11 years of age, 24 (13.3%; p = 0.18 compared with children 1–5 years of age; power = 0.21) were positive for *R. typhi* IgG and 13 (7.2%) were positive for *R. rickettsii* IgG. Eleven (85%) of 13 patient samples positive for *R. rickettsii* IgG were also positive for *R. typhi* IgG. Of the 181 samples from children 12–17 years of age, 25 (13.8%; p = 0.18 compared with children 1–5 years of age; power = 0.25) were positive for *R. typhi* IgG and 1 (0.6%) was positive for *R. rickettsii* IgG. The 1 patient sample that was positive for *R. rickettsii* IgG was also positive for *R. typhi* IgG. Thus, 62 (12%) of 513 samples tested had IgG reactive to *R. typhi,* and 20 (3.9%) had IgG reactive to *R. rickettsii.* χ^2^ analysis for trend showed no difference in *R. typhi* seroprevalence between the 3 age groups (p = 0.28; power = 0.27).

## Conclusions

On the basis of this study, ≈9%–14% of children in Nueces County have antibodies reactive to *R. typhi*. Seroprevalence appeared to increase with age. This trend did not reach statistical significance, but the power was insufficient to resolve a difference between the 3 age groups. Our results are similar to those of seroepidemiologic studies of *R. typhi* conducted in Texas and other areas of the world. Wiggers and Stewart (*5*) found that 15.7% of serum samples from an adult population in East Texas were positive for *R. typhi*.

Of the 20 samples positive for *R. rickettsii* IgG, 16 (80%) were also positive for *R. typhi* IgG and probably represent cross-reactivity, which can occur within and between the typhus fever and spotted fever groups (*6*). Because *R. rickettsii* is not endemic in South Texas, the 4 samples positive for *R. rickettsii* IgG and negative for *R. typhi* IgG may represent cases of *R. felis,* for which no test kit was available. *R. felis* can cause murine typhus–like illness, as reported, for example, in a patient from South Texas (*7*). In addition, opossums and cat fleas in South Texas demonstrate a higher infection rate with *R. felis* than *R. typhi* (*8*). It is also possible that the children in our study with test results positive for *R. rickettsii* IgG but negative for *R. typhi* IgG may have traveled outside South Texas to an area where *R. rickettsii* is endemic. They may also represent cases of infections caused by other *Rickettsia* spp., such as *R. prowazekii*, *R. parkerii*, and *R. amblyommii.*

Our study had several limitations and potential sources of bias due to the testing and sampling methods used, and these may have led to an overestimation of *R. typhi* seroprevalence. First, the reading of slides is subjective for indirect IFA assays; thus, it is possible that some negative results were deemed positive. We did not have >1 observer validate the results, but we did use a laboratory technician who was experienced at performing the tests. Second, a reciprocal titer >64 was considered positive per the test kit instructions. Use of a higher reciprocal titer for the cutoff may increase the specificity of the test and reduce the number of false-positive results. However, IgG titers decline over time (*9*), and we wanted to make sure we detected low-level titers that may have resulted from infections that occurred years ago. Third, we did not obtain medical or travel histories for the children. It is possible that some with positive test results may have had contact with other rickettsia and that their test result was positive due to cross-reactivity. Last, the convenience sample of specimens may not be representative of the Nueces County population as a whole because the specimens were not obtained through a randomized process.

Endemic murine typhus continues to occur frequently in South Texas children, as shown by the high rate of *R. typhi* seroprevalence that we found. Most cases probably go undiagnosed and spontaneously resolve. During the 1930s and 1940s, when murine typhus was more common, investigators evaluating the seroprevalence of *R. typhi* estimated that ≈700 people per year in San Antonio, Texas, were infected; whereas, the peak number of cases reported by the San Antonio Health Department in 1944 was only 91 (*10*). Physicians practicing in or near *R. typhi*–endemic areas need to consider murine typhus in the differential diagnosis of children with a febrile illness without a clear source of infection. *R. typhi* can be a cause of fever of unknown origin in hospitalized children who live in or travel to areas where this rickettsia is endemic, and it is important to know that effective antibiotic treatment is available (*3*,*4*).
